# Prevalence of K-RAS Codons 12 and 13 Mutations in Locally Advanced Head and Neck Squamous Cell Carcinoma and Impact on Clinical Outcomes

**DOI:** 10.1155/2013/848021

**Published:** 2013-04-30

**Authors:** Eric Bissada, Olivier Abboud, Zahi Abou Chacra, Louis Guertin, Xiaoduan Weng, Phuc Félix Nguyen-Tan, Jean-Claude Tabet, Ève Thibaudeau, Louise Lambert, Marie-Lise Audet, Bernard Fortin, Denis Soulières

**Affiliations:** ^1^Department of Head and Neck Surgery, Centre Hospitalier de l'Université de Montréal (CHUM), Montreal, QC, Canada; ^2^Department of Hematology and Medical Oncology, CHUM, Montreal, QC, Canada; ^3^Department of Radiation Oncology, CHUM, Montreal, QC, Canada

## Abstract

*Background*. RAS gene mutations have an impact on treatment response and overall prognosis for certain types of cancer. *Objectives*. To determine the prevalence and impact of K-RAS codons 12 and 13 mutations in patients with locally advanced HNSCC treated with primary or adjuvant chemo-radiation. *Methods*. 428 consecutive patients were treated with chemo-radiation therapy and followed for a median of 37 months. From these, 199 paraffin embedded biopsy or surgical specimens were retrieved. DNA was isolated and analyzed for K-RAS mutational status. *Results*. DNA extraction was successful in 197 samples. Of the 197 specimens, 3.5% presented K-RAS codon 12 mutations. For mutated cases and non-mutated cases, complete initial response to chemoradiation therapy was 71 and 73% (*P* = 0.32). LRC was respectively 32 and 83% (*P* = 0.03), DFS was 27 and 68% (*P* = 0.12), distant metastasis-free survival was 100 and 81% (*P* = 0.30) and OS was 57 and 65% (*P* = 0.14) at three years. K-Ras codon 13 analysis revealed no mutation. *Conclusion*. K-RAS codon 12 mutational status, although not associated with a difference in response rate, may influence the failure pattern and the type of therapy offered to patients with HNSCC. Our study did not reveal any mutation of K-RAS codon 13.

## 1. Introduction

Head and neck squamous cell carcinoma (HNSCC) accounts for 47 000 new malignancies diagnosed each year in the USA and is the sixth most common human neoplasm, representing about 3% of all cancers [1]. Despite efforts to improve conventional treatment, survival rates for these cancers have not changed significantly over the past decade.

Initial evaluation of patients includes clinical assessment, study of tumor histological characteristics and tumor grading, as well as local-regional and distant metastasis status. Traditional clinical, radiological, and histopathological characteristics are however limited in their ability to accurately predict response to treatment. This has motivated many researchers to identify molecular characteristics that may influence overall prognosis. 

A recent interest in molecular biology and genetics is motivated by the belief that understanding the origins of cancer can lead to more logical means of treating malignancies [2]. Identification of molecular events that lead to HNSCC may represent a key to predicting biological behaviour and may consequently lead to new treatment modalities that could lead to increases in survival rates. [3, 4]. Despite the recent progress in the field of molecular biology, clinicians need more tools to predict response to therapy or to identify patients at high risk of poor outcome. Identification of biological markers predictive of treatment failure would potentially permit the use of more targeted therapies or adaptation of current protocols to these patient groups in order to improve results.

Oncogenes of the *RAS* family are strongly implicated in the pathogenesis of cancer. *K-RAS* gene mutations have been reported in approximately 15–30% of human solid tumours [5–7]. This mutation is the most common abnormality of dominant oncogenes in human tumors and is a common event in the development and progression of adenocarcinomas of the pancreas (90%), colon (50%), thyroid (50%), bladder (50%), and lung (30%). The *RAS* family of genes is of particular interest in HNSCC because a mechanism for mutation (activation) of K-RAS by tobacco carcinogens has been suggested [8]. Furthermore, *RAS* mutations have been observed in other tobacco-related cancers, namely, pancreatic carcinoma and non-small cell lung carcinoma [4].

The *RAS* gene is known to encode for a family of related proteins, termed p21s, which are associated with the plasma membrane and participate in the transduction of signals involved in cellular growth and differentiation. The conversion of normal *RAS* proto-oncogenes, specifically K-RAS, to activated oncogenes is usually accomplished by point mutations involving the 12th and occasionally the 13th and 61st codons on chromosome 12. Several carcinogens preferentially bind codon 12 to create DNA adducts [9]. This results in the expression of abnormal p21 proteins harboring a single amino acid substitution favoring an active, GTP-bound state. This activates the RAS-RAF pathway and culminates in a pathologic activation of cellular mitosis.

*K-RAS* mutations are known to be associated with resistance to chemotherapy and radiation therapy, particularly in non-small cell lung and colorectal cancers [10, 11]. For metastatic cancer, the response rate to classical regimens of chemotherapy or to tyrosine kinase inhibitors is much lower in patients with the mutation. Hence, survival is lower and *K-RAS* mutations are considered a negative prognostic factor. These results have not been reproduced in HNSCC. 

A limited number of publications have examined the frequency of these mutations in the development of HNSCC. In a 1990 study published in 1990, Howell et al. [12] first described an activated RAS oncogene specific to HNSCC. Following that report, others have attempted, through different techniques, to quantify the presence of this specific mutation in head and neck cancers. While some suggested that mutational activation of RAS was not associated with the occurrence of HNSCC [2, 4, 5, 13–20], others found that *K-RAS* mutations had a direct causal role in the development of these cancers [21–23]. 

Current literature describes a low frequency of these mutations in the western hemisphere. Investigations of *RAS* mutations in the Western World have estimated the incidence of these mutations to be less than 5% [4, 13, 15–18, 20, 24]. The prevalence of this mutation increases to 18% in countries such as Spain and Taiwan [21, 25] and may be even higher in India [23, 26]. Whereas *H-RAS* mutation was detected in as much as 35% of Indian oral cancer specimens [27] and has been associated with betel nut chewing, *K-RAS* mutation prevalences vary considerably [23, 27]. Some investigators have looked at the possible association between *K-RAS *mutations and clinical correlates. The existing literature is however scarce and derived from studies with small patient numbers and wide inclusion criteria, rendering cohorts too heterogeneous for results to be interpreted. 

From these data, some authors have concluded that HNSCC with or without RAS mutations do not seem to differ clinically from each other [25]. 

Overexpression of the of the *RAS* gene product p21 in HNSCC has been reported by a number of groups, despite the low incidence of RAS mutations in head and neck cancers [28]. Abnormal expression of *RAS* genes may be attributed to mutation in the gene promoters and not to the coding region itself. Expression of this protein seems to be increased in well differentiated cancers, while its expression is low in severely dysplastic lesions and poorly differentiated cancers [29]. Authors have found a correlation between increased p21 and a more malignant and invasive biological behavior [3, 17, 28, 30–33], whereas others have correlated increased p21 expression with a favorable clinical prognosis [20, 29, 34]. In contrast, increased RAS p21 was found in poorly differentiated cancers, correlating with increased disease-free survival [34]. Oral cancers positive for *H-RAS* mutations may actually fare better than those who do not harbor, the mutation as suggested by Anderson et al. [5]. This finding, however, was not shown to be statistically significant due to the small number of positive tumors. No reference to prognosis was made by Saranath et al., whose group determined that 20 out of 57 oral tumor specimens tested positive for the mutation [27, 35].

The objectives of our study were to determine the prevalence of K-RAS codon 12 and 13 mutations, in patients with locally advanced HNSCC treated with chemoradiation therapy with or without surgery, and to evaluate the impact of these mutations on loco-regional control as well as overall, disease-free and distant metastasis-free survival at three years.

## 2. Patients and Methods

### 2.1. Patient Population

Four hundred and twenty-eight patients with stage III and IV HNSCC treated with chemoradiation therapy at Centre Hospitalier de l'Université de Montréal—Hôpital Notre-Dame and followed for a minimum of 24 months were included in this study.

### 2.2. Data Collection

Data were collected prospectively from a regular assessment of outcome variables such as response rates, local or regional recurrences, and survival rates by means of regular clinical and radiological evaluations. All patients had histological confirmation of SCC based on histological features in hematoxylin and eosin-stained tissue sections diagnosed by a pathologist experienced in head and neck pathology. 

### 2.3. Sample Preparation

Three to eight sections of 10 *μ*m were obtained from each tumor. To avoid cross-contamination during sectioning, disposable microtome blades were used, and the microtome was cleaned after cutting each specimen. The paraffin was removed by xylene and ethanol, and the tissue was then incubated in 200 *μ*L lysis buffer (10 mM Tris-HCl, pH 8.0, 1 mM EDTA, pH 8.0, 20 mM NaCl) containing 0.2 mg/mL proteinase K for 2 hours at 55°C. The mixture was then heated at 96°C for 5 minutes in order to inactivate proteinase K. Optic density was calculated for the supernatant after centrifugation of the mixture at 12000 G for 20 minutes. Four hundred nanograms of the prepared DNA were used as the template for *K-RAS* gene amplification and the remaining mixture was stored at −80°C for repeat analysis using the nested PCR technique.

### 2.4. PCR Amplification of K-RAS of Codons 12 and 13

PCR was performed in 100 *μ*L of reaction mixture containing a 400 to 500 ng of DNA, following a technique described by Hatzaki et al. [36]. Forward primer incorporated a C residue mismatch at the first position of codon 11. This created a BstNI restriction enzyme cleaving site in the amplified normal allele after PCR amplification. This cleaving site was absent in the amplified mutated DNA strand when any of the known point mutations were found on codon 12. Reverse primer incorporated a G residue mismatch at the first intron as a positive control for BstNI digestion. For codon 13, a mismatched downstream primer was used, creating a HaeIII restriction site in the wild-type allele. 

### 2.5. Digestion of PCR End Products

For codon 12, digestion was carried out with BstN1. HaeIII digestion was carried out for codon 13. Samples were then analyzed with 6% polyacrylamide gel electrophoresis. Mutated K-RAS codon 12 resulted in a 143-bp strand, whereas wild-type resulted in two strands of 114 bp and 29 bp. Mutated K-RAS codon 13 showed two strands of 85 bp and 74 bp, whereas wild-type resulted in three strands measuring 85 bp, 48 bp, and 26 bp. Positive controls for all mutations (derived from cell lines) were run with each PCR. Cell line SW480 (ATCC inc., Manassas, VA, USA) has a homozygous mutation of K-RAS codon 12. Cell line HCT116 (ATCC inc., Manassas, VA, USA) has a heterozygous mutation of codon 13.

### 2.6. Statistical Analysis

Statistical analysis was performed using Fisher's test for categorical data and Kaplan-Meier's curves and log-rank statistics for disease-free survival, overall survival, and loco-regional control.

### 2.7. Ethical Aspects

This study was approved by our institution's ethics board (reference number 09.254).

## 3. Results

All available tissue samples from 428 consecutive patients treated with chemoradiation therapy in our institution were retrieved. In total, 199 paraffin embedded biopsy or surgical specimens were recovered. DNA extraction was accomplished successfully in 197 of these. Patient characteristics did not differ statistically. Seventy-seven percent of specimens were from male subjects. Primary tumor site is listed in Table 1. Seventy-nine percent of patients initially presented with stage IV HNSCC. Chemotherapy regimens consisted of combined carboplatin and 5FU in the majority of patients (55%), and of single agent platinum salts-based drugs for the remainder of patients. K-RAS codon 12 mutations were detected in 7 of 197 DNA samples (3.5%). This value increased to 8 (4%) with the use of nested PCR techniques, suggesting an adequate sensitivity in detecting K-RAS mutations with simple PCR-RFLP. Results were reproducible, which confirmed test accuracy (data not shown). None of the samples showed mutations involving codon 13. 

No statistically significant correlation could be made between degree of histological differentiation and presence or absence of K-RAS codon 12 mutations, nor could a correlation be made between this mutation and disease stage, recurrence, or second primary tumor formation. Mutations involving K-RAS codon 12 were not more prevalent according to gender. Four of the mutations were from oropharyngeal cancers, with an even distribution between base of tongue and tonsilar lesions. The remaining three mutations were from laryngeal specimens, consisting of two supraglottic and one glottic carcinomas. 

**Figure 1:**
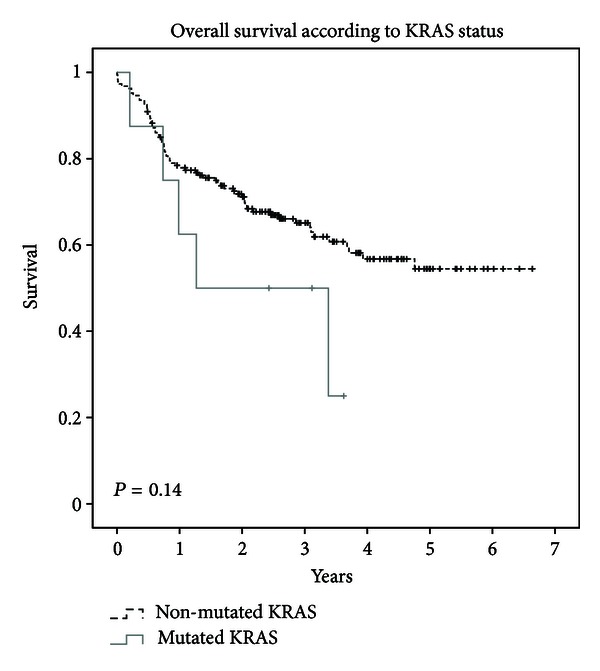
Kaplan Meier projected overall survival of wild-type K-RAS compared to mutated K-RAS.

Complete initial response to chemoradiation therapy was not influenced by mutational status. For mutated cases and nonmutated cases, complete initial response to chemoradiation therapy was 71 and 73%, respectively (*P* = 0.32). At three years, a statistically significant difference was observed for local-regional control between mutated and nonmutated cases, with respective values of 32% and 83% (*P* = 0.03). Disease-free survival was 27% and 68% (*P* = 0.12), distant metastasis-free survival was 100% and 81% (*P* = 0.30), and overall survival (see Figure 1) was 57% and 65% (*P* = 0.14) at three years. Lifetime results were not different when using the nested technique (data not shown).

## 4. Discussion

The prevalence of K-RAS codon 12 mutations in our study population is in agreement with previously published data from the western world. The PCR technique used here to detect codon 12 and 13 mutations has previously been validated in the clinical setting with a demonstration of high specificity (100%) and a detection at a low level of presence (1%) [37]. Contrary to one report, which could only identify K-RAS codon 12 mutations using a nested technique, our results indicate that simple PCR-RFLP was able to clearly identify 7 of the 8 mutated specimens studied. However, K-RAS codon 13 mutation is a very rare event in HNSCC. In a study on sinonasal carcinoma, 1% of squamous cell carcinomas harbored a K-RAS codon 12 mutations, and there were no mutations in codon 13 [9]. Furthermore, no codon 13 mutations were found in 22 SCC of the larynx in a study by Rizos et al. [18]. Also, in the study conducted by Weber et al., only one case of HNSCC out of 89 harboured a codon 13 mutation [38].

Our study does not include analysis of the two other well-known RAS oncogenes. Our estimate may therefore be conservative in that RAS gene mutations other than K-RAS may be present in our population. K-RAS gene mutations may only represent 50% of RAS mutations in head and neck cancer specimens [25]. Furthermore, K-RAS mutations themselves may have been underestimated since codon 59 and 61 were not evaluated in our study; however, mutations of these codons are even less frequent.

On the other hand, K-RAS mutations found in head and neck specimens may possibly represent mutations in the lymphocytic infiltration of the carcinoma and not the malignant epithelium itself, as was previously described by Chang et al. [39]. Blood sampling verifying this possibility was not carried out in our population. Tissue samples showing K-RAS codon 12 mutations did not show a more aggressive pattern on histology than those without the mutation.

Our study failed to determine the chronological occurrence of these mutations since all tissue specimens studied were from advanced stage III and IV cancers. All mutations were found in advanced stage IV disease and recurrences. The role of K-RAS mutations in early stages of carcinogenesis could thus not be ascertained. Whether or not K-RAS activation plays a part in early carcinogenesis remains unknown [19, 22]. Inducible activation of K-RAS in the oral cavity of mice has been objectified by Caulin et al. [22]. These tumors represent early stages of tumor progression, and their differentiation characteristics resemble those observed in benign human oral lesions. 

In our population, tumors demonstrating K-RAS codon 12 mutations did not show an increased metastatic potential compared to their nonmutated counterparts. Thyroid cancer, on the other hand, demonstrates a substantial difference in occurrence of RAS gene activation between papillary (20%) and follicular (80%) cancers, suggesting a relation between this pattern and the marked difference in metastatic potential of these cancers [40]. RAS gene activation, usually by point mutation, may be an important event in the transformation of glandular tissue to adenocarcinoma, but seems to play a lesser role in SCC formation [15].

Treatment of HNSCC has evolved over the last two decades to incorporate modalities that have resulted in decreased patient morbidity. Unfortunately, there has been little improvement in mortality rates over the same period. 

## 5. Conclusion

Although the prevalence of K-RAS codon 12 mutations is below 5% in the western hemisphere, the benefit of searching for such a mutation is considerable. Just as sarcomas represent only a fraction of laryngeal tumors and are treated surgically, HNSCC with K-RAS codon 12 mutations may represent a subset of tumors requiring special treatment considerations in order to improve outcomes. Though histology has long been accepted as the gold standard to classify tumors and orient treatment of HNSCC, molecular biology and the search for specific markers must be considered as an added tool to distinguish tumors with similar histological appearance but different behaviors. The use of biological markers may thus help overcome limitations inherent to histological classification and improve treatment outcomes by allowing the use of more specific treatment modalities. 

## Figures and Tables

**Table 1:** Patient characteristics and treatment intentions.

Characteristic	K-RAS codon 12 (no mutation)	K-RAS codon 12 (mutation)	K-RAS codon 13	All
Sex—M/F	144/44	6/1	—	150/45
Age—yr	56	62	—	56
Stage				
II	2	0	0	2
III	31	0	0	31
IV	148	6	0	154
Relapse	7	1	0	8
Site of primary tumor				
Mouth	21	0	0	21
Oropharynx	119	4	0	123
Larynx	26	3	0	29
Hypopharynx	11	0	0	11
Other	11	0	0	11
Tumor stage				
T1	21	1	0	22
T2	39	1	0	40
T3	60	1	0	61
T4	61	3	0	64
Nodal stage				
N0	21	0	0	21
N1	31	0	0	31
N2	103	5	0	108
N3	25	1	0	26
Radiotherapy				
Conventional	170	6	0	176
Adjuvant	18	0	0	18
Chemotherapy				
Cisplatin	61	1	0	62
Carboplatin	126	6	0	132
